# A hybrid algorithm for instant optimization of beam weights in anatomy-based intensity modulated radiotherapy: A performance evaluation study

**DOI:** 10.4103/0971-6203.79693

**Published:** 2011

**Authors:** Ranganathan Vaitheeswaran, Narayanan V. K. Sathiya, Janhavi R. Bhangle, Amit Nirhali, Namita Kumar, Sumit Basu, Vikram Maiya

**Affiliations:** Siemens Ltd., HealthCare Sector, Pune, India; 1 Department of Radiation Oncology, Ruby Hall Clinic, Pune, India

**Keywords:** Anatomy-based IMRT, hybrid algorithm, intensity modulated radiotherapy, optimization, fast simulated annealing

## Abstract

The study aims to introduce a hybrid optimization algorithm for anatomy-based intensity modulated radiotherapy (AB-IMRT). Our proposal is that by integrating an exact optimization algorithm with a heuristic optimization algorithm, the advantages of both the algorithms can be combined, which will lead to an efficient global optimizer solving the problem at a very fast rate. Our hybrid approach combines Gaussian elimination algorithm (exact optimizer) with fast simulated annealing algorithm (a heuristic global optimizer) for the optimization of beam weights in AB-IMRT. The algorithm has been implemented using MATLAB software. The optimization efficiency of the hybrid algorithm is clarified by (i) analysis of the numerical characteristics of the algorithm and (ii) analysis of the clinical capabilities of the algorithm. The numerical and clinical characteristics of the hybrid algorithm are compared with Gaussian elimination method (GEM) and fast simulated annealing (FSA). The numerical characteristics include convergence, consistency, number of iterations and overall optimization speed, which were analyzed for the respective cases of 8 patients. The clinical capabilities of the hybrid algorithm are demonstrated in cases of (a) prostate and (b) brain. The analyses reveal that (i) the convergence speed of the hybrid algorithm is approximately three times higher than that of FSA algorithm; (ii) the convergence (percentage reduction in the cost function) in hybrid algorithm is about 20% improved as compared to that in GEM algorithm; (iii) the hybrid algorithm is capable of producing relatively better treatment plans in terms of Conformity Index (CI) [~ 2% - 5% improvement] and Homogeneity Index (HI) [~ 4% - 10% improvement] as compared to GEM and FSA algorithms; (iv) the sparing of organs at risk in hybrid algorithm-based plans is better than that in GEM-based plans and comparable to that in FSA-based plans; and (v) the beam weights resulting from the hybrid algorithm are about 20% smoother than those obtained in GEM and FSA algorithms. In summary, the study demonstrates that hybrid algorithms can be effectively used for fast optimization of beam weights in AB-IMRT.

## Introduction

Recently, there has been a growing interest in aperture-based inverse planning (ABIP) for IMRT, as ABIP can significantly reduce the number of segments and monitor units.[1,2] This is accomplished without loss of dose coverage for the targets and with sparing of nearby critical structures. Also, IMRT plans with pre-defined anatomy-based MLC fields, known as anatomy-based IMRT (AB-IMRT), could be considered to reduce both the treatment complexity and verification burden.[3–5] The optimization of the beam weights in AB-IMRT was addressed by many investigators using different methods.[4–8] In general, the heuristic methods such as simulated annealing (SA) and genetic algorithms (GAs) are capable of escaping local optima and thus able to arrive at a global optimum.[3]

The simulated annealing method simulates the slow cooling of a sample to find low-energy states. This technique has been applied to problems in radiotherapy, especially in IMRT.[2–5] Recently several enhancements of simulated annealing method have been developed, such as parallel tempering approach.[9] In general, the method of simulated annealing can provide well-acceptable results in IMRT optimization as compared to any other optimization algorithms, mainly due to its ability to escape from the local optima.[3] However, if time is a critical factor, simulated annealing method may deliver suboptimal solutions as it employs a random search technique.[9]

On the other hand, several very efficient exact optimization algorithms have been developed in recent years.[10–12] These algorithms can now be applied to some problems of IMRT as the system sizes which can be treated are now much larger than those being treated 10 years ago. The advantage of using such exact optimization algorithms is that they take very less time as compared to iterative and heuristic algorithms. However, applying of such non-iterative methods may produce suboptimal solutions in some situations, like in those where they can get trapped into the possible local minima.

In this work, a simple and efficient optimization algorithm for AB-IMRT, called “hybrid algorithm,” is introduced in response to the drawbacks mentioned above. Our proposal is that by integrating an exact optimization algorithm with a heuristic optimization algorithm, the advantages of both the algorithms can be integrated into the created hybrid algorithm, which will lead to an efficient global optimizer solving the problem at a very fast rate. Our hybrid approach combines Gaussian elimination method (GEM) (exact optimizer) and fast simulated annealing (FSA) algorithm (a heuristic global optimizer) for the optimization of beam weights in AB-IMRT. The numerical and the clinical characteristics of the hybrid algorithm are compared with those of the GEM and FSA algorithms. In the numerical analysis, the numerical characteristics of the hybrid algorithm, such as convergence, convergence rate, consistency, number of trails and overall optimization speed, were analyzed for 8 patients in comparison with those of the GEM and FSA algorithms. The clinical capabilities of the hybrid algorithm were demonstrated in (i) prostate and (ii) brain cases.

## Material and Methods

### Anatomy-based intensity modulated radiotherapy

We used a simple anatomy-based segmentation method[3–5] for manually generated anatomy-based MLC fields in AB-IMRT. More details on how the anatomy-based fields are generated can be seen in our recent publication.[13] Figure 1 shows an example of a set of anatomy-based MLC fields for a particular beam angle.

**Figure 1a:**
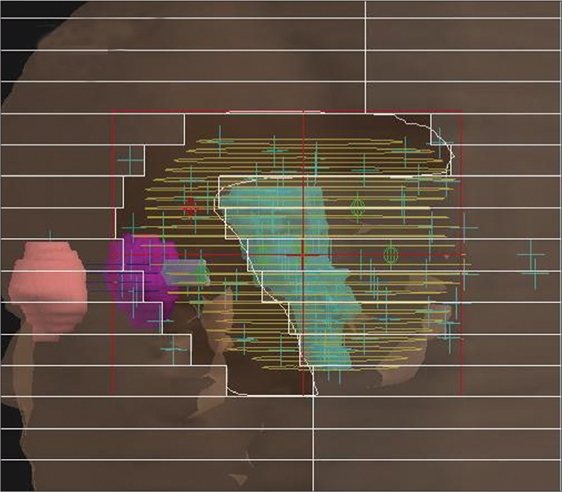
An example of a set of anatomy-based MLC fields for a particular beam angle. In the give beam angle, blocking of spinal cord present within the BEV of the target volume results in the subsequent fields as shown in Figure 1a. Also few beams directly passing through spinal cord avoiding the rest of the target volume is used in AB-IMRT plans as shown in Figure 1b in order to produce the desired dose distribution

**Figure 1b:**
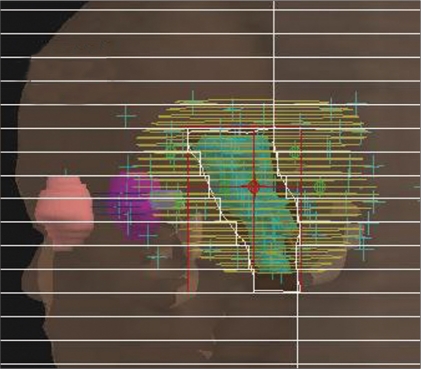
An example of a set of anatomy-based MLC fields for a particular beam angle. In the give beam angle, blocking of spinal cord present within the BEV of the target volume results in the subsequent fields as shown in Figure 1a. Also few beams directly passing through spinal cord avoiding the rest of the target volume is used in AB-IMRT plans as shown in Figure 1b in order to produce the desired dose distribution

### Hybrid algorithm

**Figure 2:**
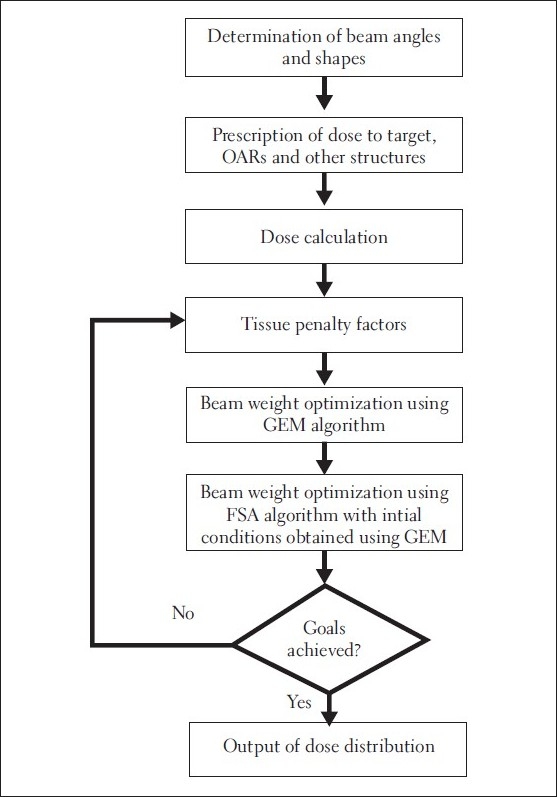
A flow chart of the proposed hybrid optimization algorithm for anatomy-based inverse planning

The proposed hybrid approach combines GEM algorithm (an exact approach) and FSA algorithm (a heuristic approach) for the optimization of beam weights in AB-IMRT using a quadratic dose-based cost function. In linear algebra, GEM is a powerful algorithm that can be used to determine the exact solutions of a system of linear equations.[14–17] Recently, the use of Gaussian elimination algorithm for optimizing beam weights in AB-IMRT has been demonstrated.[13] In our sequential optimization approach, the initial approximate solutions (beam weights) are obtained using GEM. These initial solutions are, in turn, fed into FSA algorithm for further optimization. We used MATLAB software package, which incorporates GEM and SA algorithms in its optimization tool box. In the simulated annealing (SA) optimization module, we used a fast-cooling scheme to speed up the annealing process (FSA scheme). The processes involved in the hybrid algorithm are given in the flow chart [Figure 2].

### Dose calculation

Patient contours are first generated in the CMS-XiO^®^ (4.3.1) treatment planning system, and the same system is used for dose calculation as well. In this planning system, the dose is calculated using a fast convolution superposition algorithm.[18] A dose grid size of 3 × 3 × 3 mm was used throughout the study. The slice thickness of the CT images used for planning purpose was 3 mm.

### Treatment delivery

We have tested the hybrid algorithm in patient treatments. To date, many patients have been successfully delivered AB-IMRT plans optimized using the hybrid algorithm. The AB-IMRT treatment is delivered using Siemens ONCOR Impression Plus Linear Accelerator. This linac has a facility called automatic field sequencing (AFS) with which it is possible to deliver non-IMRT treatments involving several beams and beam segments very quickly without the operator interventions. This AFS facility makes the AB-IMRT treatment delivery very fast and efficient.

### Numerical analysis

This analysis will demonstrate how the mathematical properties of the hybrid algorithm could be exploited in the optimization of single-criterion functions for AB-IMRT. To perform the analysis, different data sets (A, B, C, D, E, F, G and H) were generated that belonged to different patient cases (HandN 1, HandN 2, Brain 1, Brain 2, Abdomen 1, Abdomen 2, Pelvic 1 and Pelvic 2, respectively), representing a typical AB-IMRT planning situation. Each patient data set is represented using a quadratic dose-based cost function. The patient data sets used for the numerical analysis comprised the following:

- Beam parameters (gantry angles, number of apertures and their shapes)
- Optimization parameters (dose constraints and penalties)
- User-defined dose-control points (voxel samples)

The number of gantry angles, number of apertures per beam angle and their shapes were adapted to the anatomy of the given case. Table 1 gives the summary of the AB-IMRT plans for these cases used in the numerical analysis. Then, an attempt was made to minimize the cost functions by optimizing beam weights for each data set. The optimizations for the above-mentioned patient data sets were performed using (a) hybrid algorithm (FSA+GEM), (b) GEM and (c) FSA in order to understand the numerical abilities of the hybrid approach.

**Table 1:** Summary of AB-IMRT plan details for the cases used in the numerical analysis

*Data set*	*Patient case*	*Initial cost function ×102*	*Number of gantry angles*	*Number of apertures*	*Sampling density (points/cc)*
A	H and N 1	1.5	6	32	1.12
B	H and N 2	0.5	6	28	1.11
C	Brain 1	0.6	5	23	1.00
D	Brain 2	0.9	5	28	0.92
E	Abdomen 1	0.2	6	25	1.20
F	Abdomen 1	0.3	5	18	1.15
G	Pelvic 1	0.9	9	39	1.30
H	Pelvic 2	1.1	7	20	0.95

### Clinical analysis

In this section, we have presented a detailed account of the clinical performance of the hybrid algorithm done for 2 patient cases (prostate and brain) in comparison with GEM and FSA algorithms. The patient cases are chosen on the basis that there is a considerable geometric and dosimetric complexity involved in the planning and three dimensional conformal radiotherapy (3D CRT) is apparently not capable of producing the desired dose distribution in the cases taken for the study. In order to simplify the process of optimization, we have systematically sampled a number of dose control points in the regions of planning target volume (PTV), normal tissues and in the surrounding regions (to control spillage) only for which the dose will be calculated during optimization. A sampling density (ρ) of 1 point/cm^3^ (approximately) was used in both the patient cases. Also, a differential tissue penalty scheme was usedin both the cases in order to prioritize the goals. The PTV coverage was given the highest penalty, and the dose to the OARs was given relatively lower penalties. Moreover, the plan quality obtained using the three different algorithms were analyzed in terms of Conformity Index and Homogeneity Index. Here, the Conformity Index (CI)[19] is defined as

CI = 1 + V_n_/V_t_

where V_n_ is the volume of normal tissue receiving the prescribed dose, and V_t_ is the volume of the target receiving the prescribed dose.

The Homogeneity Index (HI)[20] is defined as

HI = [D_max_ – D_min_]/D_prescribed_

where D_min_ (dose to 2% of the PTV), D_max_ (dose to 98% of the PTV) and D_prescribed_ are the minimum, maximum and prescribed doses, respectively.

## Results

### Numerical analysis

**Figure 3:**
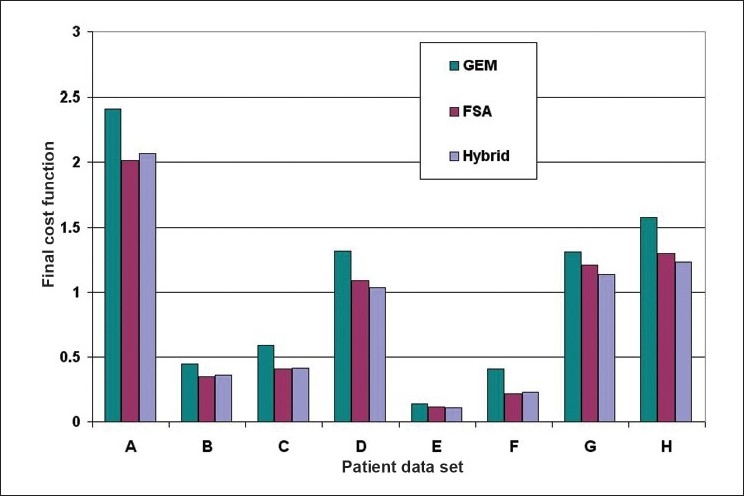
A plot of the final cost function for the patient data sets A, B, C, D, E, F, G and H in GEM, FSA and hybrid algorithms

**Figure 4:**
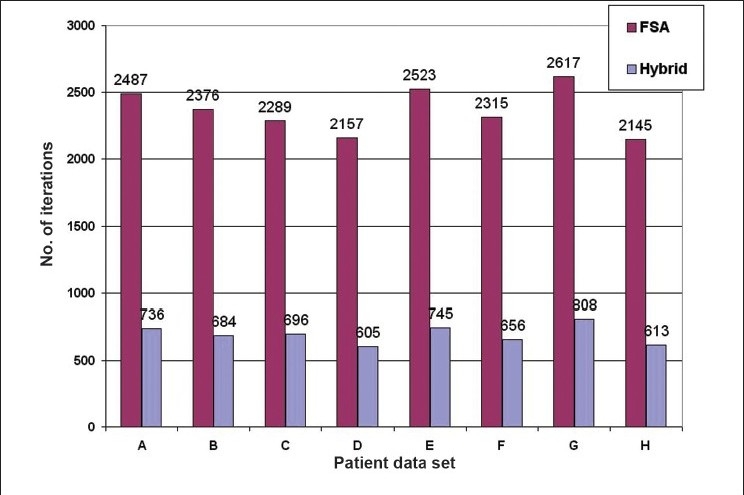
A plot of the number of iterations taken for hybrid and FSA algorithms in patient data sets A, B, C, D, E, F, G and H

**Figure 5:**
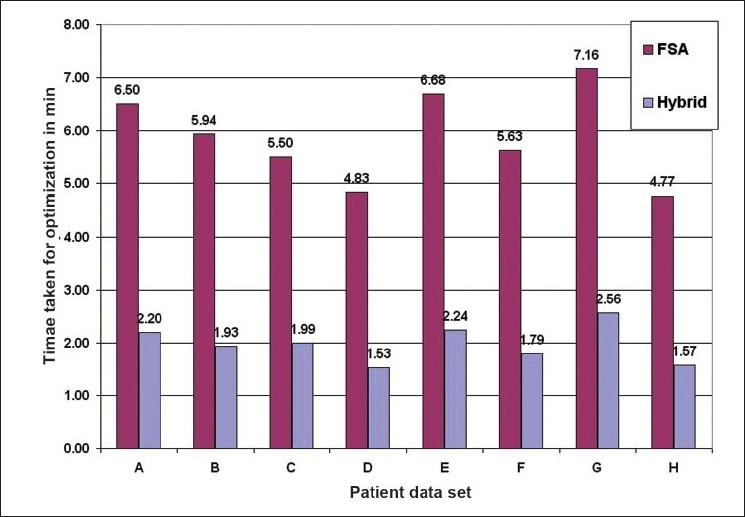
A plot of the time taken for optimization in hybrid and FSA algorithms for patient data sets A, B, C, D, E, F, G and H

The numerical analysis shows [Figures 3–5] that the convergence speed increases significantly for the hybrid algorithm as compared to the fast simulated algorithm due to the inclusion of Gaussian elimination method. Also, the consistency of the hybrid algorithm is not affected by the inclusion of Gaussian elimination method algorithm with the FSA algorithm. Most importantly, the number of iterations required to optimize the AB-IMRT problem is dramatically reduced for hybrid algorithm as compared to FSA algorithm.

The convergence is about 20% improved in hybrid algorithm as compared to that in GEM algorithm and is comparable to that in FSA algorithm in most of the cases. In our study, the convergence was measured by the percentage reduction in the cost function for a particular case. In other words, the more the percentage reduction in the cost function, the better is the convergence of the optimization algorithm. But the percentage reduction in the cost function value does not necessarily mean an equal amount of improvement in the required outcomes. However, the dose distributions obtained in the 8 patient cases used for the numerical analysis indicate that the plan with lower final cost function value appears to have apparently better dose distribution as compared to the plan with higher final cost function value. This feature is common for all the 8 patients cases included in the numerical analysis and 2 patient cases included in the clinical analysis. This observation confirms that in a single-criterion optimization, the reduction in the cost function can be used as an approximate indication of the corresponding improvement in the dose distribution. The numerical analysis also points out that an algorithm having relatively good convergence characteristics will obviously lead to better dose distribution.

### Clinical analysis

#### Prostate case

The hybrid algorithm was used to generate an AB-IMRT plan for a prostate case. In this case, we planned a dose of 57.6 Gy for the gross disease in a single phase comprising 37 fractions. The gross disease was delineated using CT images and was defined as clinical target volume (CTV). The planning target volume (PTV) was drawn with a 3-mm margin to the CTV. The volume of PTV was 160 cc. The geometry of the PTV was very complex as it was overlapping on the nearby rectum and bladder volumes. Moreover, we wanted to restrict the dose to bladder and rectum as much as possible. Because of the geometric and dosimetric complexities, we considered to execute AB-IMRT plan instead of 3DCRT plan for this case.

The summary of the treatment goals for this case is given in Table 2. Six 6-MV beams were used with 4 apertures per beam, resulting in a total of 32 beam segments. The OARs included in this case were the rectum, bladder and the two femoral heads. In order to compare the performance of the hybrid algorithm with that of GEM and FSA algorithms, respectively, we planned the same case using GEM and FSA algorithms. All plans were normalized based on the dose volume histograms (DVHs) such that 95% of the target volume was covered by the prescribed dose.

**Table 2:** Summary of treatment goals for the ABIMRT plans for prostate and brain cases

*Case*	*Structure*	*Goals*	*Tissue penalty*
Prostate case	PTV	V_57.6 Gy_ 95% and	100
		V_62 Gy <_ 55%	100
		D_max_ < 65 Gy	100
	Rectum	V_30 Gy_ < 75%	35
		V_40 Gy_ < 60%	35
		V_50 Gy_ < 40%	35
		V_60 Gy_ < 5%	35
	Bladder	V_20 Gy_ < 80%	30
		V_40 Gy_ < 60%	30
		V_60 Gy_ < 10%	30
	Femurs	D_max_ < 55 Gy	10
		V_50 Gy_ ≥ 95% and	100
Brain case	PTV	V_55 Gy_ < 55 % and	100
		D_max_ < 60 Gy	100
	Brainstem	D_max_ < 50 Gy and	45
		V_35 Gy_ < 45%	45
	Rt. Optic nerve	D_max_ < 20 Gy	20
	Lt. Optic nerve	D_max_ < 20 Gy	20
	Rt. and Lt. Eye	V_10 Gy_ < 0%	10

**Figure 6:**
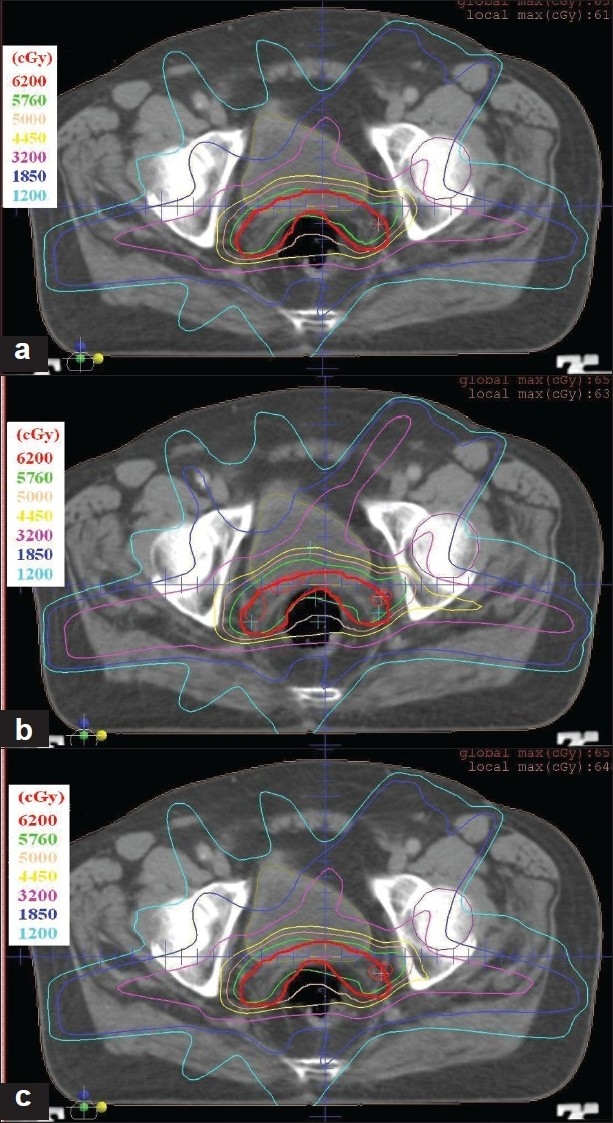
A comparison of final dose distributions in an axial slice for the prostate case obtained in (a) hybrid algorithm, (b) GEM algorithm and (c) FSA algorithm. The thick red line shows the planning target volume (PTV)

**Figure 7:**
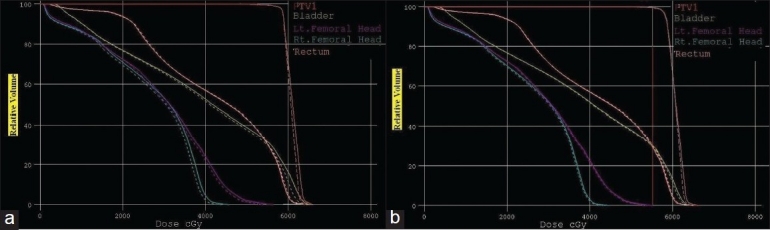
A DVH comparison for the prostate case between (a) hybrid and GEM algorithms, the solid lines denoting GEM-based plan and the dotted lines denoting hybrid algorithm–based plan; and (b) hybrid and FSA algorithms, the solid lines denoting FSA algorithm–based plan and dotted lines denoting hybrid algorithm–based plan

**Figure 8:**
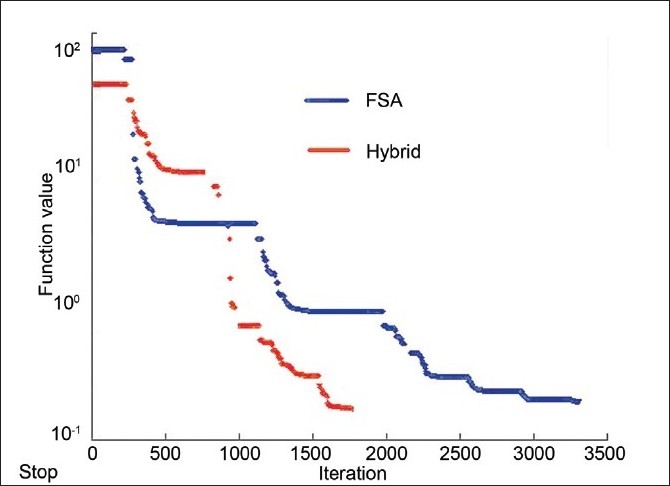
A plot of the reduction in cost function with the number of iterations for the prostate case in hybrid and FSA algorithms

**Figure 9:**
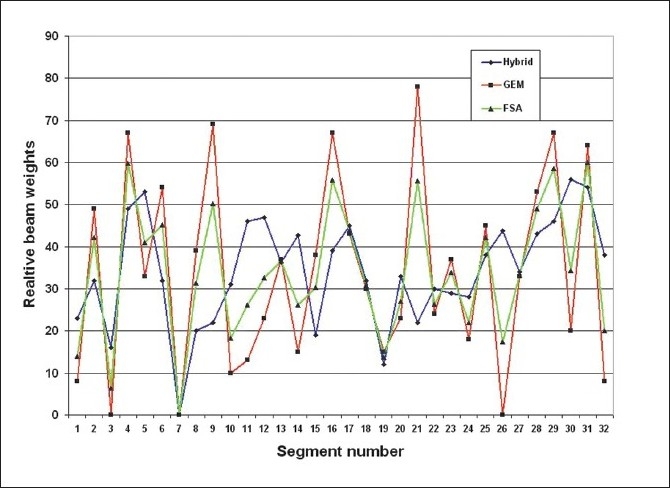
A comparison of beam weights obtained for the prostate case using hybrid, GEM and FSA algorithms

Table 3 gives the overall summary of the results obtained using hybrid, GEM and FSA algorithms. The axial dose distribution and DVHs obtained using hybrid, GEM and FSA algorithms are shown in Figures 6a and 6b, 7a and 7b. The reduction in cost function in terms of number of iterations for hybrid and FSA algorithms is shown in Figure 8. The Conformity Index (CI) for hybrid, GEM and FSA algorithms is 1.44, 1.49 and 1.47, respectively. The CI values are corresponding to the 95% dose coverage. The Homogeneity Index (HI) for hybrid, GEM and FSA algorithms is 0.25, 0.28 and 0.26, respectively. Figure 9 shows the comparison of beam weights obtained using hybrid, GEM and FSA algorithms.

**Table 3:** Summary of dose-volume indices obtained for prostate and brain cases in hybrid, gaussian elimination method and fast simulated annealing algorithms

*Case*	*Structure*	*Goals*	*Hybrid*	*Gaussian elimination method*	*Fast simulated annealing*
Prostate case	PTV	V_57.6 Gy_ ≥ 95%	95%	95%	95%
		V_62 Gy_ < 55%	22%	40%	32%
		D_max_ < 65 Gy	64 Gy	65 Gy	64 Gy
	Rectum	V_30 Gy_ < 75%	70%	70%	70%
		V_40 Gy_ < 60%	58%	58%	58%
		V_50 Gy_ < 40%	41%	41%	41%
		V_60 Gy_ < 5%	2.5%	2.4%	2.5%
	Bladder	V_20 Gy_ < 80%	78%	78%	78%
		V_40 Gy_ < 60%	51%	51%	51%
		V_60 Gy_ < 10%	10%	14%	11%
	Rt. Femur	D_max_ < 55 Gy	43 Gy	44 Gy	43 Gy
	Lt. Femur	D_max_ < 55 Gy	53 Gy	54 Gy	53 Gy
		V_50.4 Gy_ ≥ 95%	95%	95%	95%
Brain case	PTV	V_55 Gy_ < 55 %	54%	57%	57%
		D_max_ < 60 Gy	58 Gy	59 Gy	59 Gy
	Brainstem	D_max_ < 50 Gy	50 Gy	51 Gy	48.8 Gy
		V_35 Gy_ < 45%	34%	34%	32%
	Rt. Optic nerve	D_max_ < 20 Gy	15 Gy	15 Gy	15 Gy
	Lt. Optic nerve	D_max_ < 20 Gy	11.3 Gy	10.8 Gy	11.3 Gy
	Rt. Eye	V_10 Gy_ < 0%	0%	0%	0%
	Rt. Eye	V_10 Gy_ < 0%	0%	0%	0%

### Brain case

The case is a typical brain lesion of volume 308 cc (PTV). A total dose of 50.4 Gy in 25 fractions was prescribed for the PTV in this case. The sensitive normal structures such as brainstem, optic nerves and eyes were close to the target volume. Especially, we wanted to restrict the dose to the portion of the brainstem volume which was not overlapping on the target volume (rest of the brainstem), while maintaining good dose coverage to the PTV. Hence it was decided to go for AB-IMRT plan instead of 3DCRT. The summary of the treatment goals for this case is given in Table 2. Seven 6-MV beams were used with 3 apertures per beam, resulting in a total of 29 beam segments. The direct exposure to right and left eyes was avoided in the plan. The OARs included were the brainstem, right and left eye, and optic nerves. All plans were normalized based on the DVHs such that 95% of the target volume was covered by the prescribed dose.

**Figure 10:**
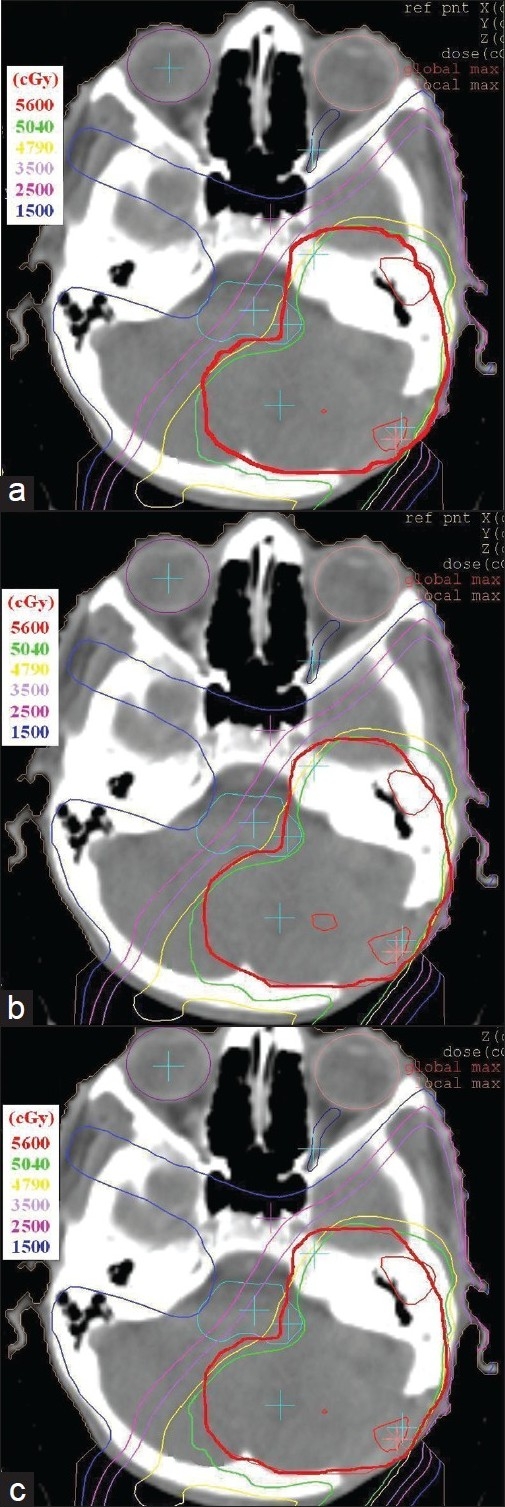
A comparison of final dose distributions in an axial slice for the brain case obtained in a) hybrid algorithm, b) GEM algorithm and c) FSA algorithm. The thick red line shows the planning target volume (PTV)

**Figure 11:**
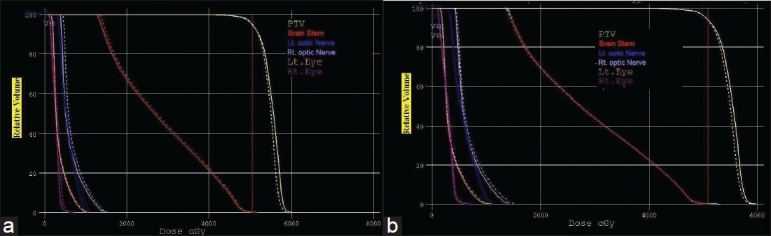
A DVH comparison for the brain case between (a) hybrid and GEM algorithms, the solid lines denoting GEM–based plan and the dotted lines denoting hybrid algorithm–based plan; and (b) hybrid and FSA algorithms, the solid lines denoting FSA–based plan and the dotted lines denoting hybrid algorithm–based plan

**Figure 12:**
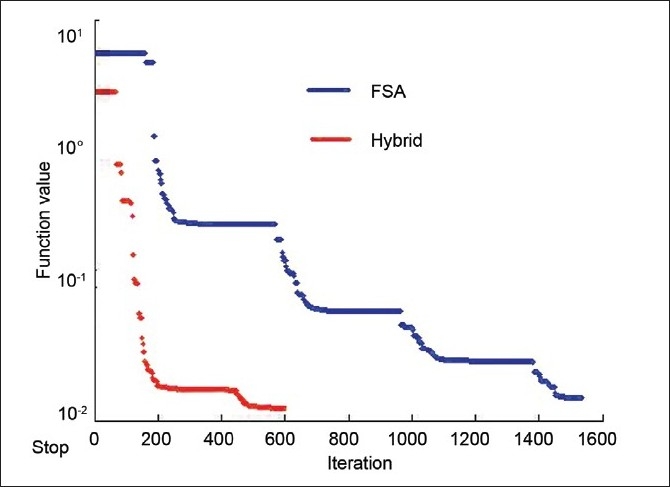
A plot of the reduction in cost function with the number of iterations for the brain case in hybrid and FSA algorithms

**Figure 13:**
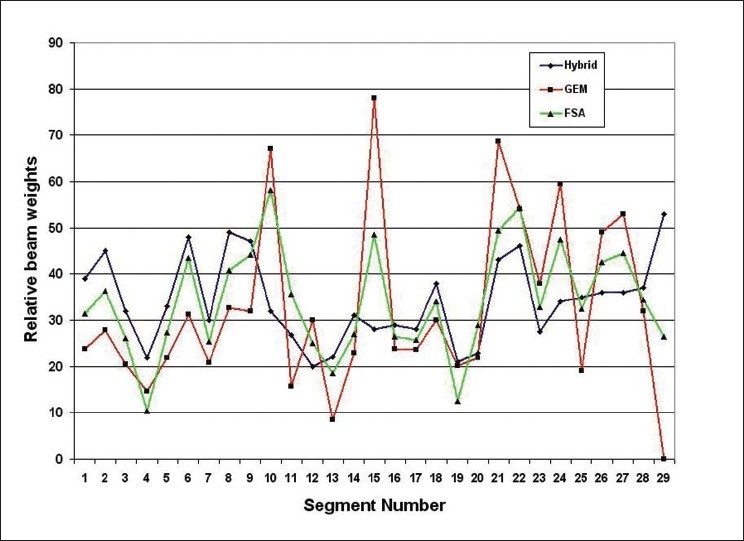
A comparison of beam weights obtained for the brain case using hybrid, GEM and FSA algorithms

Table 3 gives the overall summary of the results obtained using hybrid, GEM and FSA algorithms.The axial dose distribution and DVHs obtained using hybrid, GEM and FSA algorithms are shown in Figures 10a and 10b, 11a and 11b. The reduction in cost function in terms of number of iterations for hybrid and FSA algorithms is shown in Figure 12. The Conformity Index (CI) for hybrid, GEM and FSA algorithms is 1.37, 1.43 and 1.41, respectively. The Homogeneity Index (HI) for hybrid, GEM and FSA algorithms is 0.38, 0.41 and 0.41, respectively. Figure 13 shows the comparison of beam weights obtained using hybrid, GEM and FSA algorithms.

## Discussion

First, the comparison of the numerical capabilities of the hybrid algorithm with those of the GEM and FSA algorithms as shown in Figures 3–5 gives a clear indication that the proposed strategy (hybrid algorithm) gives a better result in terms of convergence (as compared to GEM) and convergence rate (as compared to FSA). The optimization using the hybrid algorithm is almost three times faster than that obtained using the FSA algorithm.

**Figure 14:**
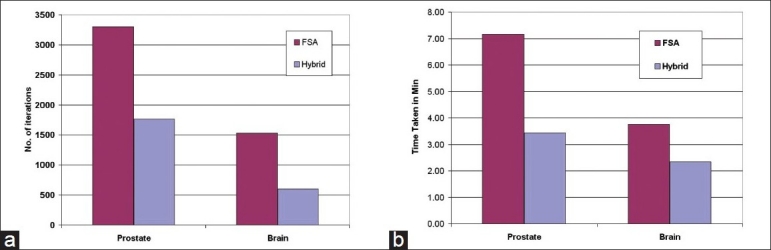
A graphical representation of the comparison of (a) the number of iterations taken for hybrid and FSA algorithms for prostate and brain cases, (b) the time taken for hybrid and FSA algorithms for prostate and brain cases

The dose distribution and DVH comparisons demonstrate that the plan obtained using hybrid algorithm offers better PTV dose conformity and dose homogeneity as compared to the plans obtained using GEM and FSA algorithms. On an average, one can observe about 2% to 5% improvement in dose conformity and 4% to 10% improvement in dose homogeneity in plans optimized using hybrid algorithm as compared to GEM- and FSA-based plans. From Figures 7 and 11 and Table 3, one can observe that the OAR-sparing is improved in the hybrid algorithm-based plans as compared to GEM-based plans. However, the plans obtained using FSA algorithm offer better OAR-sparing as compared to both GEM-based and hybrid algorithm-based plans. An impressive advantage with hybrid algorithm is the huge reduction in the number of iterations as compared to FSA algorithm [Figure 14a], as a consequence of which the optimization speed is considerably improved with hybrid algorithm [Figure 14b] in prostate and brain cases.

The plan quality obtained with hybrid algorithm is improved as compared to that obtained with GEM-based algorithm, which is due to two reasons: First, the GEM component in the hybrid algorithm gets the initial set of solutions, which when fed into the FSA component drives the heuristic process towards the goal in an effective way. Secondly, the simulated annealing component in the hybrid algorithm escapes the solution from the possible local minima during the optimization. The general observation is that the dose conformation to the tumor is very good in both the cases; and furthermore, the dose gradient in the region proximal to the OAR is steep, ensuring a good OAR protection.

**Figure 15:**
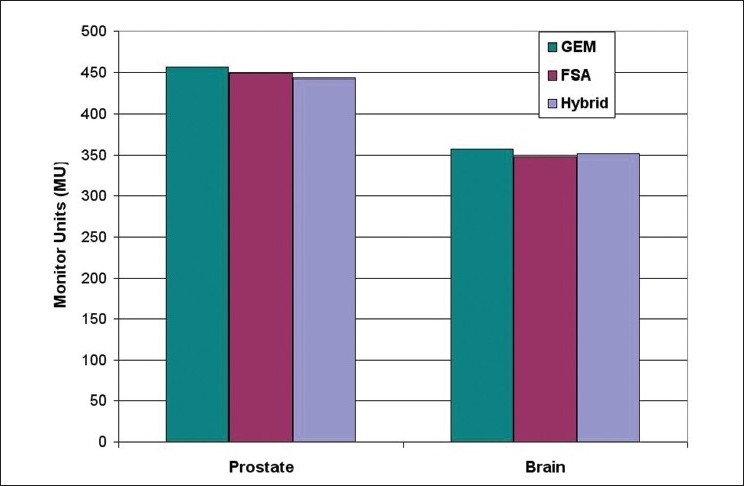
A comparison of monitor units in prostate and brain cases with respect to the optimization algorithm

Moreover, Figures 9 and 13 indicate that the hybrid algorithm is able to generate relatively smooth beam weights for the anatomy segments as compared to GEM and FSA algorithms. The smoothness of a set of beam weights obtained using an algorithm was measured using the standard deviation (SD) value of the beam weights. The larger the SD value, the greater is the variation or fluctuation in the beam weights. The SD values for hybrid, GEM and FSA algorithms were 14.1, 21.6 and 16.5, respectively, for the prostate case. Similarly, for the brain case, the SD values were 9.7, 18.6 and 12, respectively, for hybrid, GEM and FSA algorithms. Therefore, on an average, the beam weights resulting from the hybrid algorithm are about 20% smoother than those obtained from GEM and FSA algorithms. It is well known that smooth beam weights can translate into an efficient treatment delivery.[21–24] Henceit is always desirable to achieve smooth beam weights. However there is no considerable difference in the total monitor units (MUs) obtained in plans created using GEM, FSA and hybrid algorithms as shown in Figure 15.

There is an extensivedebate about whether the cost functions really possess local minima, and whether they are sufficiently close to the global minimum so that the finding of global minimum becomes insignificant.[25] However, with the available techniques to handle local minima, along withmodern fast computers, they can be invoked even in the absenceof conclusive proof of the existence of local minima.[26] The simulatedannealing technique generally guarantees global optimum in the final outcome, however with a compromise of optimization speed, which diminishes the real importance of such algorithms in a clinic. In this given situation, a methodology that helps speeding up the optimization process without degrading the quality of final solutions will be very useful in a clinic.

## Conclusion

A hybrid optimization algorithm for anatomy-based IMRT (AB-IMRT) is introduced, which integrates an exact solver (GEM) with a global optimizer (FSA) in order to get better solutions at faster rate. For the cases presented, the implemented hybrid optimizer was able to produce treatment plans comparable to FSA-based plans in terms of target coverage and OAR-sparing with a remarkable improvement in the optimization speed (about three times faster than FSA algorithm). We believe that such an improvement in optimization speed will lead to highly efficient workflow in AB-IMRT optimization, which in turn can be helpful to produce better treatment plans.
